# Gasdermin D-driven pyroptosis in sepsis: mechanisms, therapeutic strategies, and clinical translation

**DOI:** 10.3389/fimmu.2026.1801896

**Published:** 2026-04-21

**Authors:** Zixia Wang, Xiangyang Li, Bo Yuan, Junqiang Zhang

**Affiliations:** Department of Critical Care Medicine, The Second Hospital and Clinical Medical School, Lanzhou University, Lanzhou, Gansu, China

**Keywords:** gasdermin D, precision medicine, pyroptosis, sepsis endotyping, therapeutic targeting

## Abstract

Sepsis is a life-threatening organ dysfunction that leads to 11 million annual global deaths. It is characterized by severe immune dysregulation, with gasdermin D (GSDMD)-driven pyroptosis recognized as a key pathogenic mechanism. After exposure to pathogen-associated molecular patterns (PAMPs)/damage-associated molecular patterns (DAMPs), GSDMD, activated via the canonical (caspase-1) and non-canonical (caspase-4/5/11) pathways, forms plasma membrane pores, induces cell lysis, and triggers multi-organ injury. Specifically, GSDMD pores trigger lung inflammation via alveolar macrophage pyroptosis, induce hepatic high mobility group box 1 protein (HMGB1) release, perpetuate bacteremia, cause renal microthrombosis, and disrupt the blood-brain barrier. GSDMD drives both the hyperinflammatory phase (via cytokine storm, NETosis) and the immunosuppressive phase (via lymphocyte apoptosis, T-cell exhaustion), thereby defining hyperinflammatory (GSDMD-NT >120 ng/mL) and immunosuppressive (intestinal barrier failure) endotypes. Promising therapeutic agents include disulfiram (blocking Cys191 oligomerization), anti-GSDMD mAb26.5 (decreasing mortality to 30%), and the combination of imipenem and disulfiram. Clinical translation faces challenges in terms of biomarker validation, organ-specific delivery, and phase-adapted intervention. Future research directions include AI-based drug design, exosome-mediated CRISPR knockout, clinical trials on drug repurposing, and single-cell omics-integrated stratified immunotherapy.

## 1 Introduction

### 1.1 Definition and global burden of sepsis

Sepsis presents with severe dysregulation of the host immune response to infection. Based on the sepsis-3 criteria, it is defined as a life-threatening organ dysfunction. Sepsis is also characterized by a ≥2-point increase in the SOFA score, triggered by a maladaptive systemic response to pathogens (1). This condition represents a global health emergency, with recent epidemiological studies revealing 48.9 million incident cases annually worldwide. It also results in 11 million deaths, accounting for nearly 20% of the global mortality (2). The burden of sepsis exhibits striking geographic disparities. Specifically, 85% of cases are from low/middle-income countries, with mortality rates exceeding 40% in sub-Saharan Africa compared to 15%-25% in high-income nations (2). Even among survivors, 50% experience long-term cognitive impairment and 33% develop functional disabilities, leading to annual healthcare costs of more than $67 billion in the US alone (2, 3).

In addition, >60% of severe COVID-19 cases met the clinical criteria for viral sepsis due to shared pathophysiological features, including cytokine storm, coagulopathy, and multi-organ failure (4–6). The driving factors include antimicrobial resistance (affecting >40% of ICU isolates), delayed recognition (median diagnosis time >12 hours), and therapeutic limitations. Antibiotics can reduce pathogen load but fail to modulate the dysregulated host response (1, 7, 8).

### 1.2 The central role of pyroptosis in the pathogenesis of sepsis

Pyroptosis, a type of lytic, inflammatory programmed cell death distinct from apoptosis and necrosis, has emerged as the primary driver of cytokine storm and organ injury in sepsis (9–11). This process occurs through three phases: 1) inflammasome activation: Pathogen-associated molecular patterns (PAMPs; e.g., lipopolysaccharide (LPS)) or DAMPs activate cytosolic sensors (LRR- and pyrin domain-containing protein 3 (NLRP3) and absent in melanoma 2 (AIM2)) and lead to caspase-1 maturation (12, 13); 2) Gasdermin D (GSDMD) execution: Activated caspases cleave GSDMD at D275/A276 (human/mouse), thereby liberating the GSDMD-NT from autoinhibition (10, 14, 15); Importantly, GSDMD cleavage can be triggered by two distinct upstream pathways: the canonical inflammasome pathway, which activates caspase-1, and the non-canonical inflammasome pathway, in which cytosolic LPS directly engages human caspase-4/5 or murine caspase-11, leading to their activation and subsequent GSDMD cleavage (10, 16). While the canonical pathway is essential for processing pro-IL-1β and pro-IL-18 into their mature forms, the non-canonical pathway is considered a primary trigger of GSDMD-dependent pyroptosis, particularly in Gram-negative bacterial infections (17, 18). Both pathways converge on GSDMD pore formation, but the non-canonical route directly links intracellular LPS sensing to rapid pyroptotic cell death, thereby amplifying the inflammatory cascade; 3) Inflammatory amplification: GSDMD-NT oligomerizes into 10–20 nm pores within the plasma membrane, leading to ① osmotic lysis and DAMP release (HMGB1, ATP) (10, 19), ② secretion of mature interleukin 1β (IL-1β)/interleukin 18 (IL-18) through GSDMD pores (12, 20), and ③ potassium efflux. These alterations activate the NLRP3 inflammasome, forming a feed-forward loop (6, 21).

Clinical studies have reported elevated levels of pyroptosis markers (e.g., LDH, GSDMD-NT fragments) in >70% of patients with sepsis, which were correlated with sequential organ failure assessment (SOFA) scores (r=0.75) (3, 5). While eliminating intracellular pathogens (e.g., Salmonella), pyroptosis exacerbates bacteremia in polymicrobial sepsis by releasing viable bacteria from dying macrophages (6, 22). The detrimental systemic impact of excessive pyroptosis is supported by clinical evidence. Elevated plasma levels of IL-18, a key inflammatory cytokine released through GSDMD pores, have been strongly and independently associated with increased mortality in patients with severe sepsis and septic shock (23). This correlation suggests that the pyroptotic process, potentially by fueling bacteremia and systemic inflammation, is a key driver of adverse outcomes.

### 1.3 The pivotal function of GSDMD as the pyroptosis executor

**Figure 1:**
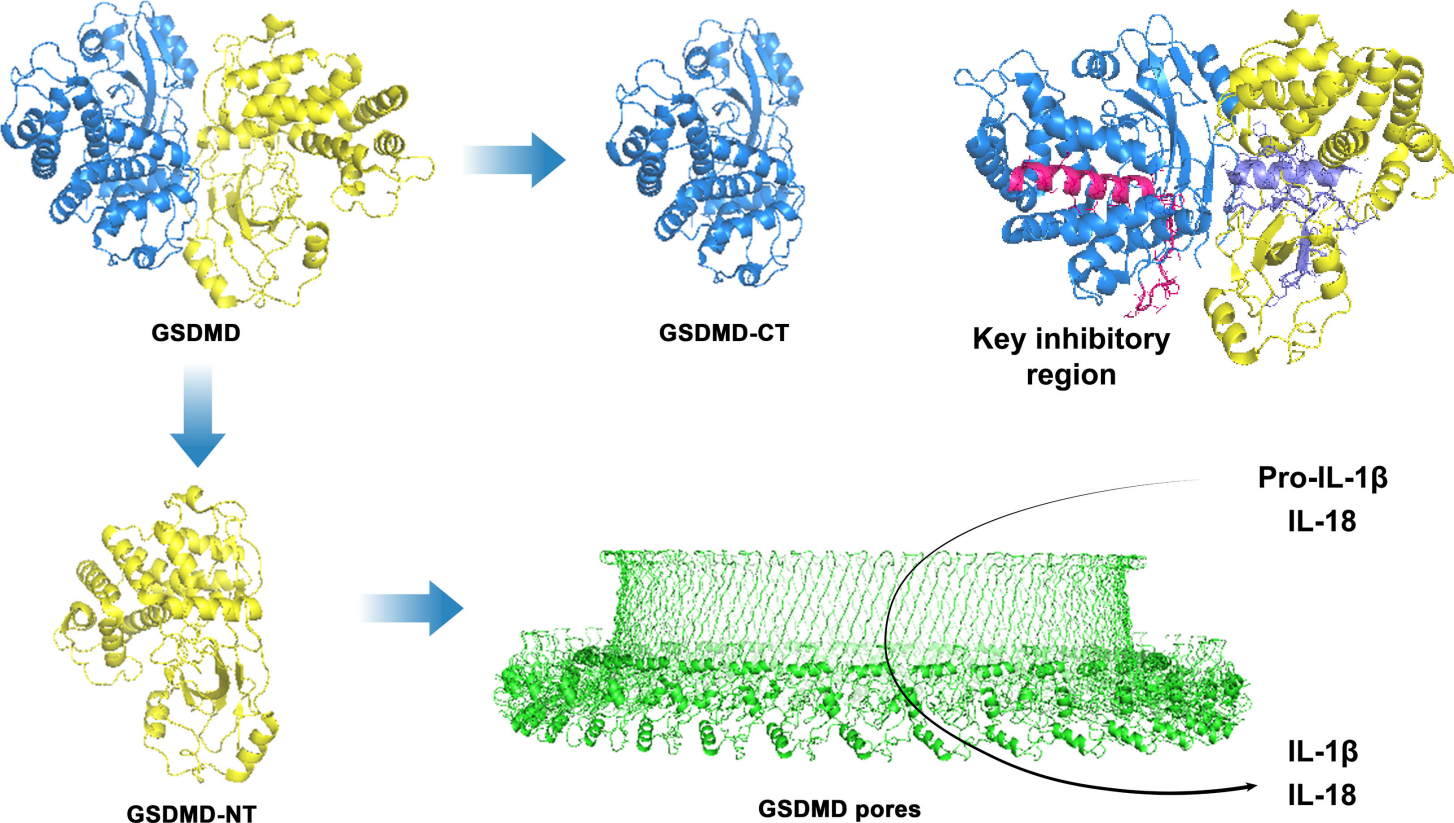
The schematic diagram illustrates the structure and mechanism contributing to GSDMD-driven pyroptosis. It consists of an GSDMD-NT and a GSDMD-CT linked by a flexible connector. The figure shows the self-inhibitory conformation, the activation switch for membrane binding by caspase cleavage, the release of GSDMD-NT at D275/A276, and selective cytokine release by oligopore assembly.

The discovery of GSDMD in 2015 established it as a non-redundant effector of pyroptosis (10, 14). The structure of GSDMD is presented in **Figure 1**. Structural studies unraveled the function of GSDMD: 1) Domain architecture: Autoinhibited conformation with GSDMD-NT and C-terminal repressor (GSDMD-CT) domains connected by a flexible linker (7, 14); 2) Activation switch: Caspase cleavage at D275/A276 leads to the release of GSDMD-NT, exposing its membrane-binding motifs. Herein, electrostatic β1-β2 loop binds to phosphatidylinositol phosphates (7), and hydrophobic α1-helix is inserted within lipid bilayers (6); and 3) Pore assembly: GSDMD-NT oligomerizes into 26–34 subunit pores with inner diameters of 10–14 nm, allowing IL-1β (17 kDa) release but excluding lactate dehydrogenase (140 kDa) until terminal lysis (6, 7, 23). Gsdmd−/− mice exhibited >60% increase in the survival rate in endotoxemia and polymicrobial sepsis models (24, 25). Specifically, endothelial-specific Gsdmd deletion reduced vascular leakage by 80%, and macrophage-specific knockout attenuated IL-1β release by 90% (8, 26).

## 2 Molecular mechanisms of GSDMD activation in sepsis

**Figure 2:**
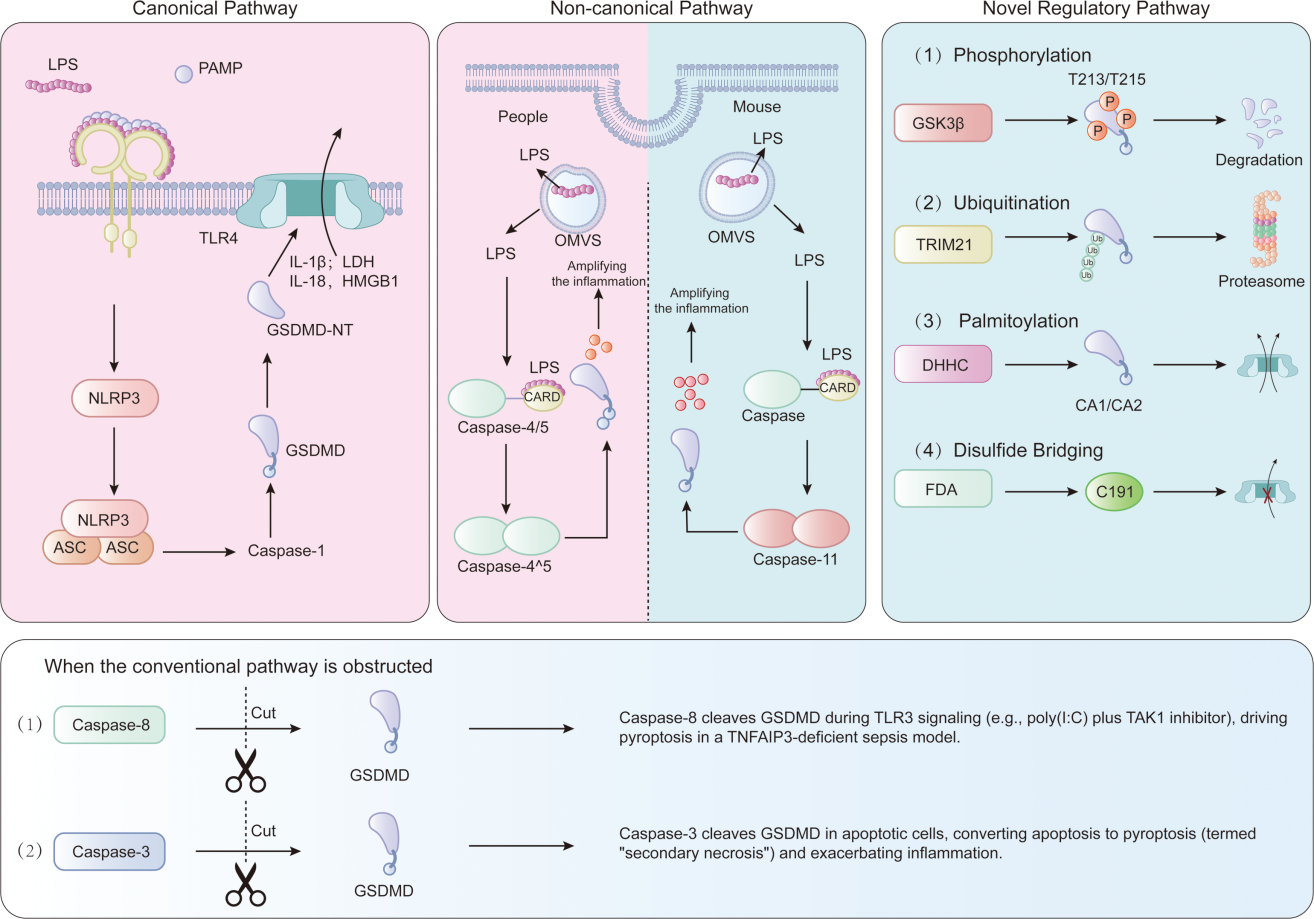
Signaling pathways and regulatory mechanisms involved in GSDMD activation in sepsis. In the canonical pathway (left), PAMPs/DAMPs (e.g., LPS, HMGB1) engage TLR4, leading to NLRP3 inflammasome assembly, ASC speck formation, and caspase-1 activation. Caspase-1 cleaves GSDMD, liberating its GSDMD-NT. In the non-canonical pathway (right), cytosolic LPS directly activates human caspase-4/5 or murine caspase-11, and these caspases subsequently cleave GSDMD. Both pathways result in GSDMD-NT oligomerization, plasma membrane pore formation, and the release of pro-inflammatory cytokines (IL-1β and IL-18) and DAMPs. Additional regulatory layers are shown, including inhibitory post-translational modifications (PTMs), such as GSK3β-mediated phosphorylation and TRIM21-mediated ubiquitination that promote GSDMD degradation, as well as DHHC7-mediated palmitoylation that facilitates pore formation. Compensatory cleavage by caspase-8 and caspase-3 can also occur in specific conditions.

Gasdermin D (GSDMD) is the executive protein in pyroptosis, a lytic inflammatory cell death pathway central to the pathogenesis of sepsis. GSDMD can be activated through canonical (caspase-1-dependent) and non-canonical (caspase-4/5/11-dependent) pathways (**Figure 2**).

### 2.1 Canonical pathway: caspase-1-dependent activation

In sepsis, PAMPs (e.g., LPS from Gram-negative bacteria) and DAMPs (e.g., HMGB1 released from dying cells) activate Toll-like receptor 4 (TLR4) (27, 28). TLR4 activation triggers the assembly of the NLRP3 inflammasome (10), which recruits the adaptor protein apoptosis-associated speck-like protein containing a CARD (ASC), thereby activatingcaspase-13 (14). Activated caspase-1 cleaves GSDMD at residue D276 (murine) or D275 (human), thereby liberating its GSDMD-NT (29, 30).

GSDMD-NT molecules oligomerize in the plasma membrane and form10–15 nm pores that disrupt ion homeostasis, lead to osmotic lysis, and facilitate the release of pro-inflammatory cytokines (e.g., IL-1β and IL-18), LDH, and alarmins (e.g., ATP and HMGB1) (31, 32). This process was found to play a critical role in macrophages and monocytes during endotoxemia and polymicrobial sepsis induced by cecal ligation and puncture (CLP) (33). Consistently, Gsdmd−/−mice revealed reduced serum levels of IL-1β, mild organ damage, and prolonged survival in LPS-induced and CLP-induced sepsis (4, 34).

### 2.2 Non-canonical pathway: caspase-4/5/11-dependent activation

Cytosolic LPS directly binds to the CARD domain of caspase-4/5 (in humans) or caspase-11 (in mice), triggering their oligomerization and autoproteolytic activation (10, 16). Activated caspase-4/5/11 cleaves GSDMD independently of TLR4 or canonical inflammasomes (17). This pathway plays a pivotal role in pyroptosis activation in endothelial cells, epithelial cells, and macrophages in sepsis caused by Gram-negative bacteria, such as E. coli (35). Caspase-11 senses cytosolic LPS delivered via outer membrane vesicles (OMVs) or HMGB1-mediated transport (20, 36). Consistently, Casp11−/−mice were more resistant to endotoxic shock, exhibiting reduced GSDMD cleavage and pyroptosis (37). Human caspase-4/5 directly cleaves GSDMD and IL-18 (but not IL-1β), thereby amplifying inflammation (18).

### 2.3 Novel regulatory mechanisms

In addition to proteolytic cleavage, several other types of post-translational modifications (PTMs) modulate GSDMD activity: 1) Phosphorylation: GSK3β phosphorylates GSDMD at T213/T215, thereby facilitating GSDMD degradation and inhibiting pyroptosis in sepsis (38); 2) Ubiquitination: TRIM21 catalyzes K48-linked polyubiquitination of GSDMD, inducing its proteasomal degradation (39); 3) Palmitoylation: DHHC7-mediated palmitoylation at C191/C192 (human/murine) facilitates GSDMD translocation to the plasma membrane and pore formation (40). Disulfiram, an FDA-approved drug, covalently modifies C191, thereby preventing pore formation and improving survival in sepsis (25).Compensatory cleavage occurs after the inhibition of canonical/non-canonical caspases: 1) Caspase-8 cleaves GSDMD during TLR3 signaling (e.g., poly(I:C) + TAK1 inhibitor), driving pyroptosis in TNFAIP3-deficient sepsis models (40); 2) Caspase-3 cleaves GSDMD in apoptotic cells, switching apoptosis to pyroptosis, also known as “secondary necrosis”, and intensifying inflammation (41).

### 2.4 Downstream effects

GSDMD pores exacerbate cellular and organ damage in sepsis through several mechanisms: 1) Release of pro-inflammatory mediators, such as IL-1β, IL-18, and LDH, through GSDMD pores (42); 2) Ion dysregulation: K+ efflux activates NLRP3, while Ca^2+^ influx triggers phospholipid scramblase (TMEM16F)-dependent phosphatidylserine (PS) exposure (43). PS enhances tissue factor (TF/F3) activity, inducing disseminated intravascular coagulation (DIC) (21); 3) Mitochondrial damage: GSDMD pores increase the permeability of mitochondrial membranes, which leads to the release of mtDNA and reactive oxygen species (ROS) (44). mtDNA activates the cyclic-GMP-AMP synthase (cGAS)-stimulator of interferon genes (STING) pathway, thereby inducing interferon β (IFN-β) production and enhancing inflammation (45). These alterations form a vicious cycle: mtDNA → cGAS-STING → IFN-β → caspase-11 upregulation → GSDMD activation (46); and 4) Extracellular vesicle (EV)-mediated propagation: Pyroptotic cells release EVs carrying GSDMD pores, inducing bystander pyroptosis in distant organs (47).

Recent studies have revealed that GSDMD exerts non-pyroptotic functions through direct targeting of mitochondria. The GSDMD-NT rapidly damages both inner and outer mitochondrial membranes by binding to cardiolipin, a phospholipid enriched in mitochondrial membranes (38, 44). This binding occurs as soon as GSDMD is cleaved, prior to plasma membrane damage, and leads to mitochondrial membrane permeabilization, loss of transmembrane potential, reduced oxidative phosphorylation, and release of mtDNA and proteins from both the matrix and intermembrane space (44). This process is independent of the B-cell lymphoma 2 family and is suppressed by genetic ablation of cardiolipin synthase or the scramblase that transfers cardiolipin to the outer mitochondrial membrane (38). The released mtDNA subsequently activates the Cgas-STING pathway, forming a positive feedback loop that amplifies inflammatory responses (45, 46).

In addition to these direct effects, recent studies have identified Ninjurin-1 (NINJ1) as the terminal executor of plasma membrane rupture (PMR) downstream of GSDMD pore formation (48, 49). While GSDMD pores (10–14 nm) permit the release of small cytokines such as IL−1β and IL−18, they are insufficient for the efflux of larger DAMPs like LDH (140 kDa) and HMGB1. NINJ1 oligomerizes into membrane−disrupting filaments, converting “hyper−permeable” cells into fully “ruptured” cells and enabling the massive release of large DAMPs that drive the cytokine storm in sepsis (48, 49). This two−step cascade—GSDMD pores followed by NINJ1−mediated PMR—amplifies systemic inflammation, and inhibiting NINJ1−dependent PMR protects against inflammasome−induced coagulation and inflammation in preclinical models (50). Thus, the GSDMD–NINJ1 axis represents a critical checkpoint for therapeutic intervention in sepsis, and dual targeting of both executors may synergistically attenuate organ injury and improve survival.

## 3 The pathogenic role of GSDMD in sepsis

GSDMD-driven pyroptosis plays a critical role in multiple organ damage in sepsis, affecting the lungs, liver, kidneys, brain and heart, highlighting the great potential of GSDMD-targeting strategies in the treatment of sepsis.

### 3.1 Organ-specific injury and systemic coagulopathy

#### 3.1.1 Lung

Sepsis-induced acute lung injury is characterized by alveolar macrophage (AM) pyroptosis subsequent to GSDMD cleavage, which triggers acute respiratory distress syndrome (ARDS). In the initial steps of this process, cytosolic LPS activates caspase-4/5 (human) or caspase-11 (murine), leading to the proteolytic cleavage of GSDMD and liberation of its GSDMD-NT (14, 51). Oligomerized GSDMD-NT forms plasma membrane pores, inducing lytic cell death in AMs and leading to the release of IL-1β, IL-18, and DAMPs. These alterations intensify pulmonary inflammation (52, 53).

Neutrophil extracellular traps (NETs) induce the overproduction of mitochondrial ROS (mtROS) in Ams. This mtROS surge acts as a critical signaling cue that directly modulates the post-translational status of NLRP3. Specifically, mtROS inhibits the ubiquitin E3 ligase, thereby suppressing the constitutive K48-linked polyubiquitination of NLRP3 that normally targets it for proteasomal degradation. Concurrently, mtROS promotes the recruitment and activation of deubiquitinating enzymes, which remove the inhibitory ubiquitin chains from NLRP3 (54, 55). Thus, NETs-derived mtROS dynamically reshapes the ubiquitination landscape of NLRP3, leading to its stabilization and assembly. The stabilized NLRP3 inflammasome then activates caspase-1, which cleaves GSDMD, thereby amplifying alveolar macrophage pyroptosis (56). This mechanistic cascade, elucidated in (56), establishes NETs not merely as static structures but as active drivers of inflammasome regulation, creating a feed-forward loop of inflammation in septic lungs.

Genetic and pharmacological inhibition of GSDMD significantly mitigated lung injury. Gsdmd-/- mice exhibited milder pulmonary edema, alveolar hemorrhage, and neutrophil infiltration subsequent to CLP or LPS exposure (52, 57). Mechanistically, GSDMD deficiency diminished IL-1β/IL-18 secretion and LDH release from AMs (52, 58). Therapeutic interventions targeting GSDMD, such as disulfiram- mediated inhibition of pore formation and wedelolactone-mediated blockade of caspase-11, attenuated bronchoalveolar inflammation, with studies reporting reduced levels of key inflammatory mediators including IL−1β, IL−18, TNF−α, IL−6 and nitric oxide (NO), as well as decreased LDH release and pyroptotic cell death in alveolar macrophages. Histopathological and functional assessments in murine models further demonstrated improved survival rates, attenuated systemic cytokine release (e.g., serum IL−1β and IL−18), and diminished bacterial burden in sepsis−induced lung injury (34, 53). Besides, targeting the upstream activators may offer clinical implications. In this regard, degrading NETs with DNase I suppressed GSDMD cleavage (56); scavenging ROS with N-acetylcysteine inhibited NLRP3 activation (56); and taurine blocked GSDMD pore assembly (58).

#### 3.1.2 Liver

Sepsis-associated liver injury (SALI) is associated with poor outcomes and increased mortality. It is clinically characterized by cholestasis and elevated serum markers of hepatocellular injury, such as alanine aminotransferase and aspartate aminotransferase. Sepsis-induced hepatic dysfunction occurs largely due to GSDMD-mediated hepatocyte pyroptosis. Cytosolic LPS activates caspase-11 (caspase-4/5 in humans), triggering GSDMD cleavage and pore formation. Membrane pore formation promotes the release of HMGB1, a DAMP that amplifies systemic inflammation by facilitating LPS entry into endothelial cells and activating the caspase-11/GSDMD pathway via a self-perpetuating cycle (35, 53, 59). Notably, hepatocyte-specific Gsdmd knockout (Gsdmd−/−) significantly attenuated liver injury in the animal models of sepsis, reduced the serum levels of alanine aminotransferase (ALT)/aspartate aminotransferase (AST), and inhibited HMGB1 release (60, 61). Hepatocyte-specific Gsdmd knockout also simultaneously suppressed ferroptosis, an iron-dependent cell death pathway characterized by mitochondrial lipid peroxidation and glutathione depletion (25, 62). These findings highlight the crucial crosstalk between pyroptosis and ferroptosis in sepsis.

Therapeutically, disulfiram was shown to inhibit GSDMD oligomerization and block membrane pore formation and HMGB1 release, thereby improving the survival of septic mice (34, 63). Bergapten enhanced mitophagy to remove damaged mitochondria, thereby inhibiting the NLRP3 inflammasome and GSDMD cleavage (34, 62). Collectively, inhibition of hepatocyte pyroptosis not only alleviates direct liver damage but also suppresses HMGB1-driven inflammation, offering a promising translational avenue against sepsis-induced hepatic failure.

Beyond its pro-inflammatory effects, HMGB1 released from pyroptotic hepatocytes also serves as an extracellular carrier for LPS. HMGB1 binds LPS and facilitates its internalization into macrophages via receptors such as RAGE or TLR4, leading to cytosolic delivery of LPS and subsequent activation of caspase−4/11 (64). This triggers GSDMD−mediated pyroptosis in macrophages, resulting in the depletion of phagocytes that are essential for bacterial clearance. Consequently, the loss of functional macrophages exacerbates bacteremia and perpetuates the septic state, creating a vicious cycle between hepatocyte pyroptosis, HMGB1 release, and macrophage dysfunction.

#### 3.1.3 Kidney

Parallel mechanisms drive sepsis-induced acute kidney injury (AKI), resulting in GSDMD-driven pyroptosis in renal tubular epithelial cells. Upon activation by caspase-1/11, GSDMD-N-terminal fragments oligomerize and form plasma membrane pores, thereby triggering lytic cell death and tubular necrosis (65). Mitochondrial dysfunction enhances this process, where GSDMD translocation induces mtROS overproduction and mtDNA release, further activating the NLRP3 inflammasome and forming a vicious cycle (52). Critically, endothelial GSDMD promotes renal microthrombosis through TF release, which initiates the coagulation cascades and enhances PS externalization by activating calcium-dependent TMEM16F scramblase (66, 67). These events accelerate fibrin deposition and renal microvascular occlusion, thereby exacerbating ischemic injury. Therapeutic strategies targeting GSDMD have shown promise in previous studies. NU6300 could bind to GSDMD oligomerization interface I, thereby preventing pore assembly and reducing tubular injury by 62% in the mice model of sepsis (68). Maresin 1 (MaR1) suppressed NLRP3 inflammasome assembly and blocked caspase-1 activation, thereby inhibiting GSDMD cleavage and IL-1β release (69). Sacubitril/valsartan blocked GSDMD processing by inhibiting the renin-angiotensin-aldosterone system (RAAS), thereby mitigating AKI severity by 45% (70). Genetic ablation of Gsdmd prevented renal microthrombi formation by 78% and improved 7-day survival from 30% to 80% in septic mice, highlighting the translational potential of targeting pyroptotic pathways.

#### 3.1.4 Brain

Sepsis-associated encephalopathy (SAE) is associated with microglial pyroptosis via GSDMD cleavage, serving as a pivotal mechanism in neuroinflammation and neuronal damage. Activation of the NLRP3 inflammasome in microglia was found to trigger caspase-1-mediated cleavage of GSDMD, leading to pore formation, IL-1β/IL-18 release, and neuroinflammation (71). Endoplasmic reticulum (ER) stress and oxidative stress (OS) enhance this process by activating the NLRP3-caspase-1-GSDMD axis (72). Cytosolic LPS activates the non-canonical caspase-11-GSDMD pathway (caspase-4/5 in humans) and exacerbates neuroinflammation through gasdermin D N-terminal (GSDMD-N)-mediated pore formation and pyroptosis (73). Microglial GSDMD also disrupts the integrity of the blood-brain barrier (BBB) by releasing DAMPs and pro-inflammatory cytokines, such as TNF-α and IL-6, thereby enhancing leukocyte infiltration and neuronal apoptosis (74).

Pharmacological or genetic inhibition of GSDMD significantly ameliorated cognitive deficits in the animal models of sepsis. Puerarin, a natural isoflavone, suppressed NLRP3/GSDMD activation, downregulated hippocampal pyroptosis, and improved learning/memory in rats subjected to CLP by downregulating caspase-1, GSDMD, and ASC (71). Disulfiram, a GSDMD inhibitor, blocked GSDMD pore formation, reduced IL-1β release, and rescued spatial memory impairment in LPS-challenged mice (75). Similarly, VX765, a caspase-1 inhibitor, and HC067047, a transient receptor potential cation channel subfamily V member 4 (TRPV4) antagonist, mitigated microglial pyroptosis and hippocampal damage and maintained cognitive function (76). After CLP, GSDMD knockout mice exhibited milder neuroinflammation, limited neuronal loss, and improved performance in the Morris water maze test(reflected by shorter escape latency, increased time spent in the target quadrant, and more platform crossings) (77). Clinical studies identified a positive correlation between GSDMD expression and SAE severity in patients with sepsis, supporting its role as a therapeutic target (78).

#### 3.1.5 Heart

Sepsis-induced myocardial dysfunction is a critical determinant of mortality in septic patients, driven by GSDMD-mediated pyroptosis in cardiomyocytes. Unlike renal tubular epithelial cells where GSDMD activation is primarily triggered by cytosolic LPS sensing via caspase-11, cardiac pyroptosis involves distinct metabolic-inflammatory crosstalk. Recent studies have elucidated that oxidative stress activates thioredoxin-interacting protein (TXNIP), which dissociates from thioredoxin and directly binds to the NLRP3 inflammasome, leading to caspase-1-dependent GSDMD cleavage and subsequent cardiomyocyte pyroptosis (79, 80). This TXNIP–NLRP3 axis is particularly prominent in cardiomyocytes due to their high metabolic activity and susceptibility to ROS during sepsis.

In addition to oxidative stress, cytosolic Ca²^+^ overload—a hallmark of septic cardiomyopathy—exacerbates mitochondrial damage by inducing mitochondrial permeability transition pore opening, resulting in loss of mitochondrial membrane potential and release of mtDNA into the cytosol (81, 82). Extracellular mtDNA functions as a DAMP that activates the cGAS-STING pathway, leading to upregulation of caspase-11 expression and further GSDMD cleavage (82). This establishes a feed-forward loop wherein mitochondrial dysfunction amplifies pyroptotic signaling. Moreover, sepsis disrupts mitochondrial dynamics by increasing expression of the fission protein Drp1 and reducing fusion protein Mfn2, promoting mitochondrial fragmentation and impaired mitophagy. Defective clearance of damaged mitochondria via the PINK1/Parkin pathway sustains ROS production and NLRP3 inflammasome activation, creating a vicious cycle that perpetuates cardiomyocyte loss (82). These unique mechanisms distinguish cardiac pyroptosis from that in other organs and highlight the need for tailored therapeutic strategies targeting metabolic stress and mitochondrial quality control.

#### 3.1.6 DIC

Beyond organ−specific injury, GSDMD−driven pyroptosis plays a pivotal role in DIC, a life−threatening complication of sepsis characterized by widespread microvascular thrombosis, multi−organ failure, and high mortality (83). Mechanistically, cytosolic LPS from Gram−negative bacteria activates caspase−11, which cleaves GSDMD and leads to pore formation (10, 16). Subsequent calcium influx activates the phospholipid scramblase TMEM16F, resulting in PS exposure on the cell surface (21, 84). Exposed PS enhances tissue TF activity, thereby promoting thrombin generation and fibrin deposition. Genetic evidence supports this mechanism. Deletion of Casp11 or Gsdmd in mice attenuates LPS−induced DIC, reduces TF activity, and prevents microvascular thrombosis (21, 84). Bacterial OMVs induce DIC through the same caspase−11–GSDMD pathway (21, 36). Pharmacological GSDMD inhibition or PS/TF neutralization also protects against DIC in experimental sepsis (85). Clinically, plasma IL−1α/IL−1β—biomarkers of GSDMD activation—correlate with PS exposure and DIC scores in septic patients (21). These findings establish GSDMD as a key link between LPS sensing and coagulation, highlighting that targeting GSDMD may simultaneously suppress inflammation and coagulopathy in Gram−negative sepsis.

### 3.2 Immune dysregulation

GSDMD-mediated pyroptosis drives biphasic immune dysregulation in sepsis, characterized by early severe inflammation and subsequent late immunosuppression. In the initial hyperinflammatory phase, pyroptotic cell death in macrophages and neutrophils leads to the release of massive amounts of IL-1β, IL-18, and DAMPs, fueling cytokine storms and systemic inflammation (51, 56). GSDMD pores amplify this inflammatory cascade and facilitate HMGB1 release (14) and mitochondrial DNA (mtDNA) extrusion, thereby activating the cGAS-STING pathway (59, 86). Crucially, GSDMD directly triggers NET formation (NETosis) by activating calcium-dependent peptidylarginine deiminase 4 (PAD4), generating prothrombotic NETs that exacerbate microvascular dysfunction (84, 87).

The transition to late immunosuppression is accompanied by GSDMD-mediated lymphocyte death and immune exhaustion. Pyroptotic macrophages release IL-18, which induces Fas ligand (FasL)-mediated apoptosis in lymphocytes (51, 88). Simultaneously, GSDMD pores in dendritic cells impair antigen presentation (89), while DAMPs from pyroptotic cells upregulate programmed death-1 (PD-1) and PD-ligand 1 (PD-L1) on T cells (14, 90). This forms an immunosuppressive milieu marked by thymic and splenic atrophy, CD4^+^/CD8^+^ T-cell depletion (88), impaired macrophage phagocytosis and cytokine production (89), and expansion of myeloid-derived suppressor cells (MDSCs) (59).

Notably, GSDMB synergizes with GSDMD to amplify lymphocyte death via caspase-4-dependent pathways (13), thereby perpetuating immune paralysis and increasing the risk of nosocomial (hospital-acquired) infections (84, 89). Emerging evidence has uncovered a critical crosstalk between GSDMD-mediated pyroptosis and NETosis in neutrophils. Non-canonical inflammasome signaling (caspase-4/11) triggers GSDMD-dependent neutrophil death, which induces neutrophils to extrude antimicrobial NETs (84, 91). Mechanistically, GSDMD is required not only for neutrophil PMR during the final stage of NET extrusion, but also for early features of NETosis, including nuclear delobulation and DNA expansion. This is mediated by the coordinate actions of caspase-11 and GSDMD in mediating nuclear membrane permeabilization and histone degradation (91). *In vivo*, DNase I-mediated dissolution of NETs during bacterial challenge increases bacterial burden in wild-type but not in Casp11 or Gsdmd mice, demonstrating the functional importance of this pathway in host defense (91).

Beyond this neutrophil-specific crosstalk, GSDMD functions as a central node within the broader PANoptosis network—a complex interplay that integrates pyroptosis, apoptosis, and necroptosis in sepsis (92, 93). The Gasdermin family, particularly GSDMD, serves as a pivotal effector facilitating communication between diverse cell death modalities (94). Inflammasome-activated caspase-1 not only cleaves GSDMD but also creates an inflammatory milieu that primes immune cells for alternative death pathways. Conversely, under conditions such as TAK1 inhibition, caspase-8 can directly engage the PANoptosome complex—a molecular platform that integrates ZBP1, AIM2, RIPK1, or NLRP12 with downstream effectors including caspase-8, RIPK3, and ASC—leading to simultaneous activation of multiple death pathways (92, 95). This intricate crosstalk establishes GSDMD as a molecular hub that integrates multiple death signals, with single-cell analyses of septic patients revealing co-expression of pyroptotic, apoptotic, and necroptotic signatures in immune cells (92, 93). Therapeutically, targeting this PANoptotic network may offer broader protection against sepsis-induced immune dysregulation than inhibiting any single pathway alone (95).

### 3.3 Sepsis endotyping

GSDMD activation patterns enable molecular subtyping of sepsis, distinguishing hyperinflammatory endotypes from immunosuppressive endotypes. The hyperinflammatory endotype​​ is characterized by high levels of GSDMD-NT in monocytes, increased levels of IL-1β/IL-18, and lactic acidosis (36, 52). Mechanistically, TLR4-TIR-domain-containing adapter-inducing interferon-β (TRIF) signaling promotes caspase-11 expression (89), while mtROS can enhance NLRP3 inflammasome assembly (34). Clinically, this process is associated with early multiorgan failure and DIC (51, 56). The plasma levels of GSDMD-NT >120 ng/mL and co-expression of GSDMD/guanylate-binding protein 2 (GBP2) in macrophages are biomarkers of the hyperinflammatory endotype (51, 88). ② The immunosuppressive endotype is characterized by GSDMD activation in the intestinal epithelium (22), T-cell exhaustion (PD-1^+^CD8^+^ >40%) (90), and microbial translocation due to intestinal barrier dysfunction. Mechanistically, intestinal GSDMD pores facilitate bacterial translocation (22), while HMGB1 expands regulatory T cells (Tregs) (14). Clinically, this process manifests as secondary infection and viral reactivation (56, 89). Fecal GSDMD-NT and CD14^+^HLA-DR^-^ monocytes >80% represent the biomarkers of the immunosuppressive endotype (22, 35).

Therapeutic stratification based on GSDMD activity shows promise. The hyperinflammatory subtype responds to the combination of anti-IL-1β biologics and disulfiram (a GSDMD inhibitor) (25, 96). The immunosuppressive subtype benefits from interferon-γ (IFN-γ) and anti-programmed death-ligand 1 (PD-L1) immunotherapy, which reverses T-cell exhaustion (14, 90). The transitional subtype may need combination therapy with caspase inhibitors and antibiotics (56, 89).

Longitudinal monitoring of GSDMD cleavage products, such as plasma GSDMD-NT, and transcriptional signatures, like the GBP2/sequestosome-1 (SQSTM1) ratio, enables dynamic endotype classification and can advance precision immunotherapy for sepsis (14, 51, 56).

## 4 Emerging therapeutic strategies targeting GSDMD

### 4.1 Small-molecule inhibitors

Small-molecule inhibitors directly target GSDMD pore formation through distinct mechanisms. Disulfiram covalently modifies Cys191/Cys192 in human/mouse GSDMD, preventing oligomerization without affecting caspase cleavage (25, 51, 97).

Necrosulfonamide and LDC7559 inhibit oligomerization by binding to GSDMD-NT, thereby blocking membrane insertion (98, 99). In the preclinical models of sepsis, disulfiram (50 mg/kg) downregulated IL-1β and LDH levels by >40%, attenuated multi-organ damage, and improved survival (25, 97). LDC7559 similarly alleviated acute lung injury by minimizing pulmonary edema and suppressing neutrophil infiltration (98). However, the use of necrosulfonamide necessitates toxicity optimization due to the off-target effects of MLKL (100).

Beyond disulfiram and necrosulfonamide, recent advances in high-throughput screening and artificial intelligence (AI)-guided drug discovery have yielded novel GSDMD inhibitors with improved specificity and distinct mechanisms of action. Using high-throughput virtual and experimental screening targeting the oligomerization interface I of GSDMD, Hu et al. identified two repurposed drugs that potently suppress GSDMD-mediated pyroptosis without modifying the Cys191 residue, thereby avoiding potential off-target effects associated with cysteine modification (34). These compounds exhibited synergistic therapeutic effects in murine sepsis models (34). More recently, an AI-screened peptide inhibitor, SK56, was developed using a deep learning-based atomic generative model (TransForPep) to target mature GSDMD-NT pores (101). Unlike conventional inhibitors that block pore formation, SK56 selectively inhibits the function of already-formed GSDMD-NT pores by engaging the cellular ESCRT membrane repair system. In delayed-treatment sepsis models, SK56 improved mouse survival even when administered after disease onset, highlighting its therapeutic potential for clinical scenarios where patients present after pyroptosis has already been initiated (101).

### 4.2 Biologics

Biologics offer high-specificity inhibition of GSDMD but face delivery challenges. Anti-GSDMD mAbs (e.g., mAb26.5) bind to the pore-forming domain of GSDMD, sterically hindering oligomerization (17, 99), while siRNA nanoparticles downregulate GSDMD expression via liver-targeted lipid carriers (17, 97). In mice with sepsis, mAb26.5 reduced the plasma levels of HMGB1/KIM-1 and improved the survival rate from 20% to 70% (99), whereas siRNA nanoparticles achieved 80% GSDMD knockdown in the liver and suppressed systemic inflammation (35, 97). The development of humanized mAbs and macrophage-targeted siRNA delivery remains a key therapeutic goal.

### 4.3 Upstream inhibition

Indirect strategies target the upstream activators of GSDMD or mitochondrial stress. NLRP3 inhibitors, such as MCC950 and OLT1177, block inflammasome assembly and prevent caspase-1 activation and GSDMD cleavage (102, 103). For instance, MCC950 prevented organ injury in sepsis models (102), while OLT1177 showed promising effects in Phase II trials on gout (103). Mitophagy inducers, such as urolithin A, markedly damaged mitochondria and inhibited mtDNA release and caspase-11 activation (62). In septic mice, urolithin A reduced TNF-α levels by 45% and improved survival by enhancing mitochondrial quality control (62).

### 4.4 Combination therapy

Combining GSDMD inhibitors with antibiotics can decrease pathogen load and suppress inflammation. Antibiotics, such as β-lactams, lyse bacteria and enhance the release of PAMPs that exacerbate pyroptosis (97, 104), while GSDMD inhibitors, such as disulfiram, block secondary inflammation. In E. coli-mediated sepsis models, imipenem + disulfiram reduced bacterial burden 100-fold and decreased mortality from 90% to 30%, outperforming monotherapy (97, 104). This synergistic strategy highlights the potential for clinical translation in polymicrobial sepsis.

## 5 Challenges in clinical translation

The transition from preclinical findings to the clinical application of GSDMD-targeted therapies faces multifaceted challenges. Specifically, biomarker validation, organ-specific delivery, disease heterogeneity, and long-term safety remain key hurdles.

### 5.1 Validation of biomarkers

Validation of serum GSDMD or its cleavage products (e.g., GSDMD-N) as diagnostic/prognostic markers remains challenging. Although elevated levels of GSDMD-N were found to be correlated with sepsis severity and organ damage in patients (e.g., higher levels in non-survivors vs. survivors) (21), standardized assay methods for clinical application are lacking. Enzyme-linked immunosorbent assay (ELISA)-based methods exhibited variability in detecting circulating GSDMD fragments due to low abundance and rapid clearance (105). Moreover, GSDMD activation occurs in several cell types (e.g., macrophages, endothelia), undermining its specificity as a biomarker. Combining GSDMD-N with traditional markers, such as lactate and procalcitonin, may improve the predictive value (21); however, large-scale cohort studies are still needed for validation (106).

### 5.2 Organ-specific delivery

Targeting GSDMD in specific cell types, such as macrophages and endothelial cells, necessitates advanced delivery systems. Nanocarriers functionalized with cell-specific ligands, such as CD44 receptor-targeted peptides, have shown promise in murine models of sepsis. For instance, liposomes conjugated with CD44-binding hyaluronic acid selectively delivered GSDMD inhibitors (e.g., disulfiram) to activated macrophages, thereby mitigating lung injury (107). Similarly, endothelial-targeted nanoparticles loaded with caspase-11 siRNA mitigated vascular leakage (10). Notably, the efficacy of such systems is highly dependent on the disease stage. They are likely most effective during the early hyperinflammatory phase of sepsis, when widespread endothelial activation and increased vascular permeability may paradoxically facilitate nanoparticle access to target tissues. However, physiological barriers (e.g., endothelial glycocalyx degradation in sepsis) limit the extravasation of nanoparticles (4). Optimizing the size (<50 nm) and surface charge (neutral) of nanoparticles can enhance their tissue penetration, but rigorous toxicity profiling is still needed (4).

Recent breakthroughs in nanomedicine have enabled organ−specific delivery of nucleic acids and inhibitors. Lipid nanoparticles have been successfully employed to co−deliver mRNA and siRNA for *in situ* macrophage engineering; surface modification with targeting peptides such as CRV enables selective uptake by macrophages, achieving efficient gene silencing and protein expression in septic tissues (108). Similarly, liposomes decorated with cRGD peptides exploit “neutrophil hitchhiking”—binding to circulating neutrophils that naturally home to inflamed sites—thereby enhancing drug accumulation in primary infection foci and reducing systemic inflammation (109). Beyond synthetic carriers, biomimetic nanoparticles such as macrophage membrane−coated nanoparticles loaded with miRNA mimics or inhibitors have been shown to selectively deliver cargo to lung macrophages, attenuating pyroptosis and acute lung injury in sepsis models (110). These platforms also enable combination strategies, such as co−delivery of antibiotics and GSDMD blockers, to simultaneously tackle infection and inflammation (108).

### 5.3 Disease heterogeneity

The progression of sepsis involves dynamic phases, including early hyperinflammation (pyroptosis-dominant) and subsequent late immunoparalysis. Therefore, stage-specific interventions are critical: ① In the hyperinflammatory phase, GSDMD inhibitors (e.g., necrosulfonamide) may limit IL-1β/IL-18 release and organ damage in LPS/CLP models (111, 112); ② In the immunoparalytic phase, prolonged GSDMD blockade may impair bacterial clearance. In Gsdmd−/− mice, delayed clearance of Klebsiella pneumoniae increases the risk of mortality (9), highlighting the need for timed therapy (113). Stratifying patients by their immune status (e.g., HLA-DR expression on monocytes) may guide the timing of intervention (28).

### 5.4 Safety concerns

Long-term inhibition of pyroptosis may compromise the immune response against pathogens, and the results will be: ① Increased susceptibility to infections: Gsdmd−/− mice showed more severe Salmonella infection due to reduced macrophage pyroptosis (17); ② Compensatory cell death pathways: GSDMD inhibition may enhance apoptosis/necroptosis and exacerbate tissue damage (76). However, short-term blockade of GSDMD (e.g., 3-day treatment with disulfiram) in CLP models prolonged survival without increasing bacterial load (96).

The following strategies may help mitigate the risks of immune compromise: ① Temporary inhibition: Controlled-release nanoparticles can be applied for transient GSDMD suppression (107); ② Cell-type-specific targeting. Neutrophil-specific Gsdmd deletion was shown to worsen sepsis (13), whereas macrophage-specific Gsdmd knockout exhibited protective effects, emphasizing the need for precision targeting (114).

## 6 Future perspectives

### 6.1 Precision medicine approaches

Single-cell RNA sequencing (scRNA-seq) of immune cells from patients with sepsis enables cell-type-specific mapping of GSDMD activation. This approach can help identify high-risk subpopulations, such as monocytes with elevated GSDMD cleavage or hyperactive caspase-4 (24, 62). Spatial transcriptomics can resolve organ-specific pyroptosis “hotspots” (e.g., lung endothelial cells or hepatic macrophages) and guide targeted drug delivery (20, 36, 115). Liquid biopsies detecting circulating GSDMD-NT fragments or pyroptosis-derived DAMPs (e.g., HMGB1, IL-18) can serve as dynamic biomarkers for monitoring treatment (53, 116). Furthermore, machine learning can help integrate multi-omics data (scRNA-seq, proteomics) to predict patient-specific responses to GSDMD inhibitors (e.g., disulfiram (24), necrosulfonamide (16)), thereby optimizing the design of clinical trials assessing the effects of novel nanotherapies or monoclonal antibodies (115).

### 6.2 Advanced delivery systems

Exosome-mediated CRISPR-Cas9 delivery is a promising strategy for myeloid-specific GSDMD knockout to suppress pyroptosis in sepsis (117, 118). Engineered exosomes loaded with CRISPR-Cas9 components (sgRNA targeting GSDMD and Cas9 mRNA) can be functionalized with myeloid-specific surface ligands (e.g., CD11b antibodies) to selectively target macrophages and monocytes (119, 120). This approach minimizes off-target effects and enhances the efficiency of delivery to phagocytic cells in inflamed tissues (121). Preclinical studies have shown that exosome-delivered CRISPR-Cas9 downregulates GSDMD expression by >80% in the mouse model of sepsis, attenuating pyroptosis, IL-1β release, and multi-organ damage (103, 120).

Optimization of exosome loading capacity and *in vivo* stability represents the key challenges (122). Innovations, like electroporation-enhanced cargo loading and hybrid exosome-liposome nanoparticles, can improve the encapsulation of CRISPR components (118, 121). Future studies should address manufacturing scalability and the risks of immune clearance through PEGylation or CD47 “don’t eat me” signal engineering (116, 122). Validation of the results in large animals with sepsis and monitoring of off-target editing via whole-genome sequencing are necessary before clinical translation (103, 120).

### 6.3 Clinical pipeline updates

The clinical translation of GSDMD inhibitors for treating sepsis is advancing. Disulfiram leads the pipeline since it covalently targets GSDMD-C191 to block pyroptosis. Preclinical studies have shown that disulfiram can significantly improve survival in mice with LPS-induced sepsis by inhibiting GSDMD pore formation and subsequent release of inflammatory cytokines, such as IL-1β and IL-18 (16, 123). A phase II trial (NCT ID pending) has been designed to evaluate the efficacy of disulfiram in human sepsis. The trial focused on patient stratification based on the cleavage products of circulating GSDMD (GSDMD-N) and inflammatory biomarkers, such as IL-18 and LDH (88, 124). In addition to disulfiram, novel candidates, like NU6300 (targeting GSDMD oligomerization interface I) (88), maresin 1 (suppressing NLRP3/GSDMD via Nrf2) (125), and 4-octyl itaconate (4-OI, inhibiting the caspase-4/11-GSDMD axis) (126), have shown synergistic potential in the mouse models of sepsis. Dose adjustment remains necessary to prevent off-target effects, such as disulfiram-mediated inhibition of aldehyde dehydrogenase (16). Furthermore, combination therapy (e.g., NLRP3 inhibitors + GSDMD blockers) is needed to target upstream inflammasomes (125). Real-time monitoring of GSDMD activation biomarkers in clinical trials is crucial for assessing target engagement (127).

### 6.4 Interdisciplinary synergy

The convergence of AI, structural biology, and pharmacology represents a paradigm shift in targeting GSDMD-mediated pyroptosis in sepsis. AI-driven tools, like AlphaFold3, can help predict the conformational dynamics of GSDMD at the atom level, assessing its GSDMD-NT and autoinhibitory interactions (128). These models can accurately simulate GSDMD oligomerization and membrane insertion mechanisms, revealing cryptic binding pockets for designing inhibitors (129). Virtual screening leverages the results of these predictions to identify compounds targeting critical sites, such as the β1-β2 loop or oligomerization interfaces, thus blocking pore formation (7). For example, in silico docking identified disulfiram and necrosulfonamide as covalent inhibitors that sterically hinder the membrane translocation of GSDMD-NT (24). Machine learning optimizes hit compounds by predicting ADMET properties and refining binding affinity (130).

Integration of multi-omics data—such as transcriptomics and proteomics derived from patients with sepsis—with AI facilitates the identification of novel GSDMD regulators and patient-specific therapeutic targets (115). This approach helped identify biomarkers (e.g., elevated caspase-4/11 activity) to stratify patients who will likely benefit from GSDMD-targeted therapy (53). Cross-disciplinary collaboration accelerates in silico-to-*in vivo* translation. Particularly, this approach can facilitate the advancement of AI-predicted inhibitors to preclinical validation in sepsis models (131). Future efforts should focus on the real-time adaptation of treatment using patient-derived data, thereby paving the way for personalized treatment strategies targeting pyroptosis in sepsis (28, 132).

## 7 Conclusion

Targeting GSDMD is critical for disrupting the self-amplifying cycle of inflammation in sepsis. GSDMD-driven pyroptosis orchestrates severe inflammation in the early stage of sepsis through cytokine storm (IL-1β/IL-18), NETosis, and mtDNA-cGAS-STING activation, while late-stage pore formation induces immunosuppression via lymphocyte depletion and T-cell exhaustion. Cross-disciplinary collaboration is needed to transform this mechanistic insight into clinical breakthroughs: ① Precision endotyping can leverage GSDMD-NT/caspase-4 signatures to stratify hyperinflammatory and immunosuppressive subsets; ② Therapeutic innovation lies in the combination of covalent inhibitors (e.g., disulfiram), CRISPR-Cas9 silencing, and ligand-functionalized nanocarriers for cell-specific delivery; and ③ Dynamic biomarker panels can help validate plasma GSDMD-NT + mtDNA + IL-18 for real-time monitoring of treatment. This framework, bridging molecular pathology, stratified interventions, and biomarker-monitored adaptation, can help break the vicious cycle of organ failure and immune paralysis, and advance toward personalized anti-pyroptotic regimens to prevent the mortality and long-term morbidities of sepsis.
